# Implementation of Public Funded Genome Sequencing in Evaluation of Fetal Structural Anomalies

**DOI:** 10.3390/genes13112088

**Published:** 2022-11-10

**Authors:** Po Lam So, Annie Shuk Yi Hui, Teresa Wei Ling Ma, Wendy Shu, Amelia Pui Wah Hui, Choi Wah Kong, Tsz Kin Lo, Amanda Nim Chi Kan, Elaine Yee Ling Kan, Shuk Ching Chong, Brian Hon Yin Chung, Ho Ming Luk, Kwong Wai Choy, Anita Sik Yau Kan, Wing Cheong Leung

**Affiliations:** 1 Department of Obstetrics and Gynecology, Tuen Mun Hospital, Hong Kong SAR, China; 2 Department of Obstetrics & Gynaecology, Prince of Wales Hospital, Hong Kong SAR, China; 3 Department of Obstetrics & Gynaecology, Queen Elizabeth Hospital, Hong Kong SAR, China; 4 Department of Obstetrics & Gynaecology, Pamela Youde Nethersole Eastern Hospital, Hong Kong SAR, China; 5 Department of Obstetrics & Gynaecology, Queen Mary Hospital, Hong Kong SAR, China; 6 Department of Obstetrics & Gynaecology, United Christian Hospital, Hong Kong SAR, China; 7 Department of Obstetrics & Gynaecology, Princess Margaret Hospital, Hong Kong SAR, China; 8 Department of Pathology, Hong Kong Children’s Hospital, Hong Kong SAR, China; 9 Department of Radiology, Hong Kong Children’s Hospital, Hong Kong SAR, China; 10 Department of Paediatrics, Prince of Wales Hospital, Hong Kong SAR, China; 11 Department of Paediatrics and Adolescent Medicine, Li Ka Shing Faculty of Medicine, The University of Hong Kong, Hong Kong SAR, China; 12 Clinical Genetics Service Unit, Hong Kong Children’s Hospital, Hong Kong SAR, China; 13 Prenatal Genetic Diagnosis Centre, Department of Obstetrics & Gynaecology, Chinese University of Hong Kong, Hong Kong SAR, China; 14 Prenatal Diagnostic Laboratory, Tsan Yuk Hospital, Hong Kong SAR, China; 15 Department of Obstetrics & Gynaecology, Kwong Wah Hospital, Hong Kong SAR, China

**Keywords:** fetal structural anomalies, genome sequencing, diagnostic yield, clinical impact

## Abstract

With the advancements in prenatal diagnostics, genome sequencing is now incorporated into clinical use to maximize the diagnostic yield following uninformative conventional tests (karyotype and chromosomal microarray analysis). Hong Kong started publicly funded prenatal genomic sequencing as a sequential test in the investigation of fetal structural anomalies in April 2021. The objective of the study was to evaluate the clinical performance and usefulness of this new service over one year. We established a web-based multidisciplinary team to facilitate case selection among the expert members. We retrospectively analyzed the fetal phenotypes, test results, turnaround time and clinical impact in the first 15 whole exome sequencing and 14 whole genome sequencing. Overall, the molecular diagnostic rate was 37.9% (11/29). De novo autosomal dominant disorders accounted for 72.7% (8/11), inherited autosomal recessive disorders for 18.2% (2/11), and inherited X-linked disorders for 9.1% (1/11). The median turnaround time for ongoing pregnancy was 19.5 days (range, 13–31 days). Our study showed an overall clinical impact of 55.2% (16/29), which influenced reproductive decision-making in four cases, guided perinatal management in two cases and helped future family planning in ten cases. In conclusion, our findings support the important role of genome sequencing services in the prenatal diagnosis of fetal structural anomalies in a population setting. It is important to adopt a multidisciplinary team approach to support the comprehensive genetic service.

## 1. Introduction

Fetal structural anomalies are common and affect about 2–3% of pregnancies [1]. Babies born with congenital anomalies can have significant morbidity and mortality [2]. The underling etiologies are complex. In the past, many epidemiologic studies and research have been performed to investigate the causes leading to congenital malformations [3]. With the recent advances in massive parallel sequencing technology, genetic disorders have been revealed to have an important contribution to congenital anomalies [4]. Two large prospective studies using whole exome sequencing to evaluate pregnancies with unselected fetal structural anomalies on prenatal ultrasound and negative karyotype and chromosomal microarray analysis (CMA) reported a diagnostic genetic variant rate of 8.5–10% [5,6]. Subsequently, a number of similar cohort studies and meta-analyses of these studies were published, confirming their diagnostic value [7,8,9].

Therefore, a 2022 updated position statement published by the International Society for Prenatal Diagnosis (ISPD) recommends the prenatal genome-wide sequencing is beneficial for (1) a current pregnancy with a fetus with a single major anomaly or with multiple organ system anomalies and non-diagnostic CMA or no CMA result, suggesting of a possible genetic etiology by clinical genetic expert review; (2) a prior undiagnosed fetus (or child) affected with a major single or multiple anomalies and the recurrence of similar unexplained anomalies in the current pregnancy [10]. The American College of Medical Genetics and Genomics (ACMG) also supports the consideration of its use when a diagnosis cannot be obtained using routine prenatal methods in a fetus with one or more significant anomalies [11]. Currently, prenatal exome sequencing is the main approach used for genome-wide sequencing in clinical practice. Genome sequencing, which has potential in the detection of variants outside the protein-coding exome and other forms of structural variations, is now emerging [12,13,14,15,16].

The prenatal genetic diagnosis can provide parents with more information about the health and prognosis of their fetus, including the functional anomalies and developmental outcomes apart from the physical anomaly. This information is important for informed decision-making in reproductive choice, pregnancy management, postnatal care and expanded reproductive options in subsequent pregnancies [17]. Several studies were published demonstrating the usefulness of prenatal exome sequencing in clinical management that exceeded the diagnostic yield [18,19,20,21,22,23,24]. However, the optimal use of this comprehensive molecular diagnostic technology in public clinical service is lacking.

In April 2021, the Hong Kong Hospital Authority launched a publicly funded next-generation sequencing (whole exome sequencing (WES) and whole genome sequencing (WGS)) as a sequential test in the investigation of fetal structural anomalies in all pregnant women. The aim of the new program was to enhance our prenatal diagnosis for special prenatal cases with the presence of fetal ultrasound anomalies after receiving a negative finding by conventional tests (CMA and karyotyping). The objective of the current study was to evaluate the one- year clinical performance as well as the clinical utility of WES/WGS in public funded service to identify the genetic diagnosis for undiagnosed fetal structural anomalies.

## 2. Material and Methods

### 2.1. Study Population and Selection

This was a retrospective review of all women who had undergone publicly funded genomic sequencing for investigation of ultrasound fetal abnormalities between April 2021 and March 2022. The study was approved by the Central Institutional Review Board of the Hospital Authority (reference: PAED-2022-013).

In 2021, the Hong Kong population size was about 7,000,000, with 23,485 deliveries among our eight public hospitals with obstetric services (Kwong Wah Hospital, Queen Elizabeth Hospital, Queen Mary Hospital, Pamela Youde Nethersole Eastern Hospital, Princess Margaret Hospital, Prince of Wales Hospital, Tuen Mun Hospital and United Christian Hospital) under the Hospital Authority, Hong Kong SAR Government. Each of these eight hospitals had a prenatal diagnostic clinic that provided prenatal genetic screening and diagnosis services to women residing nearby. For pregnant women with fetal ultrasound abnormalities confirmed by the maternal-fetal medicine specialists in the clinic, they were offered invasive prenatal diagnostic testing. The standard workflow consisted of quantitative fluorescent polymerase chain reaction (QF-PCR) for rapid common aneuploidies detection, and if QF-PCR showed normal results, CMA was performed to look for copy number variations.

Since April 2021, all pregnant women were eligible for publicly funded whole exome or genome sequencing during pregnancy or after a fetal demise or pregnancy termination when they had one or more fetal malformations with uninformative QF-PCR and CMA likely to have an underlying genetic etiology, evaluated by a multidisciplinary team. The multidisciplinary team, with the abbreviation FMPRG, was made up of 15 experts in the following specialties: Foetal Medicine (8), Pathology (1), Radiology (1), Genetics (3) and the Laboratory (2). Detailed prenatal phenotyping with ultrasound was done by fetal-medicine specialists in prenatal diagnostic clinics. Additional fetal assessment by fetal magnetic resonance imaging (MRI) was performed in some cases, depending on the available expertise in different hospitals. The upper gestational limit for termination of pregnancy in Hong Kong is 24 weeks. Further relevant information on the terminated or demised fetuses was gained at postmortem examination if couples agreed. The case summary with anonymous clinical information and radiological and pathological data/images were prepared by case managers in different units and put in the web-based platform via the webmaster for discussion among the panel members. This online platform, which was established by the Department of Pathology of Hong Kong Children’s Hospital, could facilitate data sharing and multidisciplinary discussion. For any case submission, the web master notified all panel members via email. The testing criteria of the National Health Service (England) fetal whole exome service was used as a reference for our panel case selection. Patients were considered eligible for exome or genome sequencing if their fetus had a single major anomaly or multiple organ system anomalies on ultrasound suggestive of a possible genetic etiology after a multidisciplinary review with more than 7 members voting for the test before a pre-set deadline. The selected cases would have the genomic sequencing carried out by one of the two laboratories: the Prenatal Diagnostic Laboratory at Tsan Yuk Hospital using trios WES and the Prenatal Genetic Diagnosis Centre at the Chinese University of Hong Kong using trios WGS (PMID: 35441720). Each laboratory received fetal samples from the obstetric units in its catchment area. Written informed consent was obtained from the parents after pre-test counseling with verbal and written information given by the fetal-medicine specialists. The information provided included test limitations, possible test results (such as negative, positive, and uncertain genetic variants) and their implications. Incidental findings, ACMG 59 secondary findings and carrier status for autosomal recessive conditions were also included in the pre-test consent procedure for WGS, with an opt-out option. The genomic sequencing was performed on DNA extracted from amniocytes, chorionic villi, placental tissue, cord blood, or skin biopsy. Parental peripheral blood samples were obtained for trio analysis. Maternal cell contamination was excluded in the fetal DNA using QF-PCR data of the fetus and the mother. The turnaround time was proposed to be 4 calendar weeks for ongoing pregnancies and 4 calendar weeks after further investigation results are available (e.g., postmortem report, babygram) for fetal demised/terminated cases.

### 2.2. WES Procedure and Variant Interpretation

Genomic DNA was subjected to exome enrichment using double-stranded DNA capture baits from Twist Comprehensive Exome kit (Twist Bioscience, San Francisco, CA, USA), which targets approximately 36.8 Mb of the human coding exome regions. The generated library was sequenced on Illumina NextSeq 500 (Illumina, San Diego, CA, USA) to obtain at least 30× coverage depth for >98% of the targeted bases. Sequencing data were analyzed using the Congenica^®^ analytical pipeline, which included read alignment to reference genome (GRCh37), variant calling (single nucleotide and small deletion/insertion variants), and variant annotation. Variant selection and prioritization were evaluated based on provided clinical information, suspected mode of inheritance and other laboratory data. Targeted regions were defined as coding exons +/− 5 bp. Only variants in the targeted region with clear gene-phenotype evidence from Genomics England PanelApp and OMIM and a minor allele frequency (MAF) < 1% reported in the Genome Aggregation Database were investigated. Disease-causing variants reported in ClinVar, DECIPHER, and Mastermind were also considered. Pathogenicity interpretation of genetic variants was based on the ACMG variant classification guidelines. Only variants in genes related to the patient’s phenotype were reported.

### 2.3. WGS Procedure and Variant Interpretation

Genome sequencing was based on a PCR-free library constructed with genomic DNA extracted from the sample. Paired-end sequencing was performed on a next-generation sequencing platform (NovaSeq, HiSeq X Ten or MGISEQ-2000). A minimal 30-fold read depth was generated for each case unless otherwise indicated. Sequencing reads were aligned to the reference genome (GRCh37/hg19) using the Burrows-Wheeler Alignment tool (PMID: 19451168). This test comprehensively identified genomic variants, including single-nucleotide variants (SNVs), small insertions and deletions (InDels), copy-number variants (CNVs), structural rearrangements (SVs), and absence of heterozygosity (AOHs). SNV/InDels calling was carried out by HaplotypeCaller (GATK) and annotated by ANNOVAR (PMID: 20601685) with different public reference databases. SNV/InDels interpretation was performed by referencing the medical literature and online databases according to the guidelines of the ACMG. For CNV analysis, reads were classified into sliding windows (50 kb with 5 kb increment) in terms of the aligned coordinates. After GC correction and population-based normalization, CNV detection was performed by an in-house pipeline (Increment Rate of Coverage), and interpretation was carried out by referencing the medical literature and online databases according to the guidelines of ACMG. For analysis of structural variants, uniquely aligned read-pairs were processed for identifying the chimeric read-pairs supporting a potential translocation, inversion, insertion or complex rearrangement. For AOH detection, genotyping was carried out by analysis of the mpileup file (Samtools). Regions (>5 Mb) with a decreased rate of heterozygous SNVs and an increased rate of homozygous SNVs were detected as regions with AOH. Clinically significant SNVs/InDels, CNVs, SVs and regions with AOHs related to patients’ clinical phenotype were reported. In addition, incidental findings, ACMG 59 secondary findings and carrier status for autosomal recessive conditions were reported if parents’ consent was obtained.

### 2.4. Post-Test Follow-Up

The post-test counseling was carried out by trained genetic health professionals such as maternal-fetal medicine specialists with genetic expertise from the respective prenatal diagnostic clinic and/or clinical geneticists from two university hospitals (Queen Mary Hospital and Prince of Wales Hospital), Hong Kong SAR Department of Health or Hong Kong Children’s Hospital. This included the disclosure of genetic results, explanation of the molecular diagnosis if found, further possible genetic tests, pregnancy management options, neonatal care and implication for other family members. The parents were also informed about recurrence risk and options for future family planning, including prenatal or pre-implantation genetic testing. The follow-up information, including pregnancy outcomes, was put back into the platform for educational and research purposes.

### 2.5. Study Data Collection and Analysis

We collected data from the case summaries in the online platform and laboratory databases. We studied the clinical phenotypes, the diagnostic yield, test turnaround times and the clinical impacts of the genetic results. The clinical phenotypes included prenatal phenotypes seen on fetal imaging (i.e., ultrasound and MRI) and postnatal phenotypes or postmortem reports. The diagnostic yield was defined as the percentage of the number of cases with pathogenic/likely pathogenic variants identified via WES/WGS considered to have caused the fetal structural anomaly from the total number of selected cases in a year. We also collected the number of variants of uncertain significance (VUS) as well as incidental and secondary findings for those who consented to reporting. Turnaround time was measured as days between the time that the test was ordered with patient consent received by the laboratory until the return of the final written diagnostic test report. For terminated cases, the turnaround time was counted from when the postmortem report was available. We also assessed the impact of this new clinical service on clinical management by sending out a survey to case managers to collect data about reproductive choice, clinical decision-making, pregnancy management and outcome, and neonatal treatment. Descriptive statistical tests were performed using SPSS (Windows version 23; IBM Corp, Armonk, NY, USA).

## 3. Results

### 3.1. Cohort Characteristics

**Figure 1:**
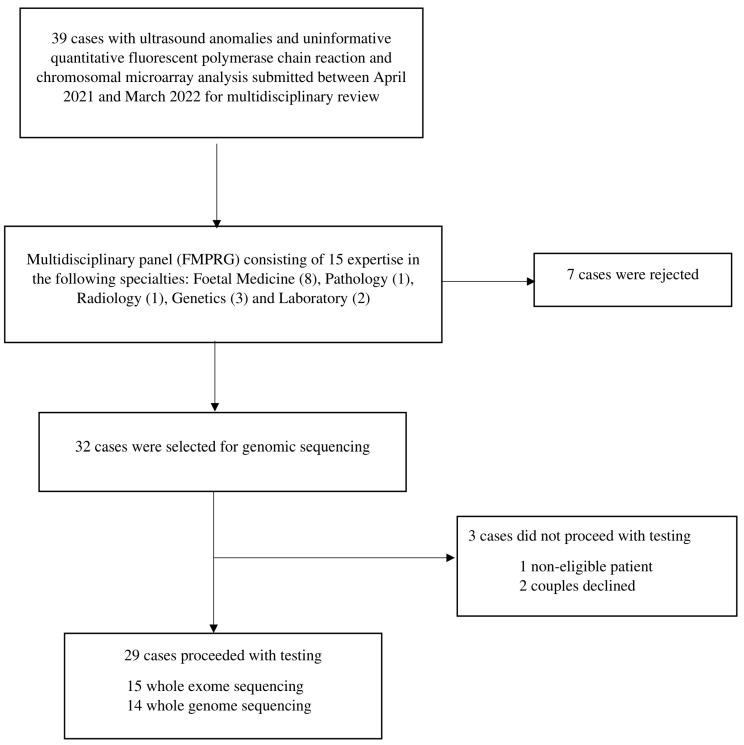
Flowchart of the genome-wide sequencing service.

From April 2021 to March 2022, 39 patients with fetuses that had structural anomalies were reviewed by the multidisciplinary team, and 32 fetuses were selected for the high probability of monogenic disorders (Figure 1). Three patients were excluded because two patients declined further genetic testing after counseling, and one patient was not entitled to the public funded test due to being a non-local citizen. The overview characteristics of 29 patients are included in Table 1. The majority were Chinese patients (89.7%), and 62.1% were tertiary-educated. Almost all were married (96.6%) with two consanguineous relationships and had planned pregnancies (79.3%); 65.5% of them were nulliparous. Three patients had conceived through assisted reproductive technologies. There were 28 singletons and one twin pregnancy. The median maternal age was 33 years (range, 25–39 years). Two had a pregnancy history affected by a genetic condition, including a chromosome 6 abnormality and alpha thalassemia major. One had a family history of Down’s syndrome (second- and third-degree relatives).

The prenatal phenotypic information of all cases was obtained from a prenatal ultrasound scan. In addition, five fetuses were investigated with fetal MRI, which supported the ultrasound finding of brain anomalies. The ultrasound structural abnormalities included multisystem anomalies (11/29, 37.9%), single system anomalies (11/29, 37.9%), non-immune hydrops fetalis (6/29, 20.7%) and fetal growth restriction (1/29, 3.4%).

Six patients (20.7%) had genomic sequencing before 24 weeks of pregnancy, two (6.9%) during ongoing pregnancy after 24 weeks, one (3.4%) after intrauterine death and 20 (69.0%) after the termination of pregnancy. All patients and their partners were sequenced as complete parental-fetal trios. WES was performed on 15 trios, and WGS was performed on 14 trios. The median gestational age for sequencing requests during ongoing pregnancy was 19^+5^ weeks (range, 15–32^+1^ weeks), and the median turnaround time was 19.5 days (range, 13–31 days). For the remaining 21 pregnancies with termination or stillbirth, the median turnaround time was 23 days (range: 14–56 days).

### 3.2. Overview of Diagnostic Yield and Prenatal Ultrasound Identified Abnormalities

Among the selected cases with WES/WGS performed, diagnostic genetic variants (pathogenic/likely pathogenic) associated with prenatal phenotypes were identified in 11 cases (11/29, 37.9%). Autosomal dominant disorders accounted for 72.7% (8/11), autosomal recessive disorders for 18.2% (2/11), and X-linked disorders for 9.1% (1/11). All autosomal dominant disorders were caused by de novo variants, and all autosomal recessive and X-linked disorders were caused by inherited variants. The twelve contributing diagnostic variants included three nonsense, four missense and five frameshift variants. VUS were detected in five cases (5/29, 17.2%), and there were incidental findings in two cases (2/14, 14.3%) and secondary findings in three cases (3/14, 21.4%). Two novel genetic variations predicted as pathogenic in *NEB* and *KMT2D* were discovered. The former was a nonsense variant, and the latter was a frameshift variant associated with a premature stop codon downstream. Both were likely to result in protein loss resulting from a loss-of-function allele.

Of the structural anomalies detected on prenatal ultrasound, the diagnostic yield was 100% (1/1) in fetuses with isolated skeletal abnormalities, 57.1% (4/7) in fetuses with isolated brain abnormalities, 36.4% (4/11) in fetuses with multiple organ systems (more than one fetal organ system abnormality) and 33.3% (2/6) in fetal hydrops. No molecular diagnosis was made in cases with isolated renal malformations, isolated facial malformations, and intrauterine growth restriction.

### 3.3. Overview of Pregnancy Outcome and Clinical Impact (Table 2 and Table 3)

#### 3.3.1. Pregnancy Outcome

Among the eight patients with WES/WGS ordered during ongoing pregnancy, one pregnancy ended in miscarriage at 18 weeks, one pregnancy was terminated, and the remaining six pregnancies resulted in a live birth, including one case of monochorionic diamniotic twin pregnancy continued the pregnancy after selective feticide.

#### 3.3.2. Clinical Impact- Helped Reproductive Decision-Making

Of the six patients with WES/WGS performed to help decision-making regarding continuation or termination of pregnancy before 24 weeks of gestation, one received a definite molecular diagnosis and ended up in miscarriage (case ES6); one received VUS result and continued the pregnancy (case ES7); four received negative results (cases GS8, GS9, GS10 and GS11) in which one underwent pregnancy termination based on ultrasound finding (case GS10). In case ES6, the fetus had multiple anomalies and hydrops fetalis. The parents opted to wait for the WES result. This case ended in a miscarriage after 2 weeks before the availability of the genetic result. WES detected a de novo pathogenic variant in the *HRAS* gene confirming Costello syndrome (OMIM 218040). In case ES7, cystic hygroma and hydrops fetalis were identified. A maternal inherited VUS was detected in the *LZTR1* gene associated with both autosomal dominant Noonan syndrome 10 (OMIM 616564) and autosomal recessive Noonan syndrome 2 (OMIM 605275). The mother attended a genetic clinic where she was not found to have any features of Noonan syndrome. Since the public variant databases had conflicting interpretations of its pathogenicity and no prenatal phenotype of fetal hydrops was reported in the literature, it was classified as VUS. The fetus had resolution of hydrops at 22 weeks of gestation. The parents decided to carry on with the pregnancy. The baby was born alive at 38 weeks with clinical features of Noonan syndrome and was referred to the genetic clinic for assessment and follow-up. This genetic finding facilitated the parents in making a reproductive choice and the subsequent neonatal management. Case GS11 was a monochorionic diamniotic twin pregnancy with cystic hygroma and hydrops detected in one of the fetuses in the first trimester. Chorionic villus sampling was performed. After normal CMA and karyotyping results, the parents decided selective feticide by radiofrequency ablation to minimize the potential neurological damage to the normal co-twin. Subsequent negative WGS results supported the parental decision to continue the pregnancy of the phenotypically normal co-twin. The baby was born alive at 41 weeks and in good condition. In case GS10, a megacystis, bilateral dilated renal pelvises, umbilical cord cyst and oligohydramnios were identified. Vesicoamniotic shunting was performed. After the negative WES result, the parents decided to terminate the pregnancy because of the poor prognosis. Overall, the uncertain or negative genetic result contributed to the decision to continue the pregnancy in four cases.

#### 3.3.3. Clinical Impact- Guided Perinatal Management

In two patients with WGS performed to guide perinatal management, one with inherited molecular diagnosis had implications in neonatal management and future reproductive counseling (case GS1), and one with an uncertain result had an adjustment in pregnancy management (case GS6). In case GS1, hydrocephalus, periventricular nodular heterotopia, mega cisterna magna and partial dysgenesis of the corpus callosum were detected at 28 weeks of gestation. WGS was performed on the umbilical cord blood sample saved at birth. A maternal inherited pathogenic variant was found in the *FLNA* gene, which was associated with X-linked periventricular nodular heterotopia (OMIM 300049). A paternal inherited *HBA* gene deletion of the alpha-thalassemia trait was also detected. Postnatal MRI brain of the female newborn confirmed the prenatal ultrasound findings. The infant had normal growth and development at 8 months old. The family was referred to the genetic clinic for further work-up of family members and counseling on reproductive planning for future pregnancy. In case GS6, aortic stenosis, aortopulmonary window, malformation of cortical development and early onset fetal growth restriction were identified at 21 weeks of gestation. A maternal inherited VUS was detected in the *MAP2K2* gene, which was associated with the autosomal dominant cranio-facio-cutaneous syndrome (OMIM 115150). The mother was phenotypically normal. In the absence of pathogenic findings, fetal growth was closely monitored. Subsequent fetal MRI brain showed no structural abnormalities. The pregnancy ended in live birth at 35^+2^ weeks of gestation when abnormal fetal Doppler occurred. At birth, the baby was admitted to the neonatal intensive care unit because of uncontrolled heart failure, with surgical ligation of the patent ductus arteriosus. Aortic stenosis and secundum atrial septal defect were noted. At 13 months old, the child showed no dysmorphic features but gross motor delay.

#### 3.3.4. Clinical Impact: Aided Future Family Planning

Of the 21 patients with WES/WGS performed to aid future family planning, they had significant fetal structural anomalies resulting in termination of pregnancy and intrauterine fetal demise before the testing request. Postnatal phenotyping by external examination was available in 19 cases, while the final full autopsy performed by pathologists was available in 11 cases to support the prenatal structural findings. Nine pregnancies were found to be harboring pathogenic/likely pathogenic variants that contributed to the phenotype observed on prenatal imaging and/or autopsy. Seven of them had de novo mutations (*RARB*, *PPP1R12A*, *NIPBL*, *COL1A1*, *SMARCB1*, *RERE*, *KMT2D*). The genetic finding provided the underlying genetic cause of the fetal anomalies and supported counseling the parents about low recurrence risk for their future pregnancy. The remaining two fetuses had mutation variants (*NEB*, *ASPM*) of an autosomal recessive disorder transmitted from their parents. The diagnosis assisted specific counseling in the future recurrence risk of one in four, and the parents could use the result to pursue preimplantation genetic diagnosis in a future pregnancy. Three patients received inherited VUS results (cases GS3, GS4 and GS7) and two of them also received inherited incidental findings (cases GS3 and GS7), which might have implications for future pregnancies. In case GS4, semilobar holoprosencephaly, bilateral microphthalmia, absent left lens, hypotelorism, bilateral cleft lip and cleft palate, low thoracic scoliosis, suspected hemivertebra and bilateral club foot were detected by ultrasound at 13^+6^ weeks. A paternal inherited VUS in the *GLI2* gene was also detected in addition to a likely pathogenic variant in the *RERE* gene. Defects in the *GLI2* gene can cause autosomal dominant holoprosencephaly (OMIM 610829) and Culler-Jones syndrome (OMIM 615849). Further work-up for the husband, as well as family cascade screening of the husband’s family, could help to delineate the significance of the VUS, which was suggested by the clinical geneticist. In case GS3, agenesis of the corpus callosum and small cerebellum was detected by ultrasound at 21^+3^ weeks. One likely pathogenic paternally inherited variant in the *LAMC3* gene, together with another maternal inherited VUS in-trans arrangement affecting the same gene, was identified by WGS. Bi-allelic *LAMC3* mutations can cause autosomal recessive occipital cortical malformations (OMIM 614115). However, the fetal anomalies seen on ultrasound were already explained by another pathogenic variant in the *SMARCB1* gene. Therefore, the pathogenicity of the VUS in the *LAMC3* gene was still uncertain. In case GS7, hydrops fetalis and bilateral pleural effusion were found at 20 weeks of gestation. The parents were related as first-degree cousins, and this was their first pregnancy. WGS detected two incidental findings, two VUS and the absence of a heterozygosity region. The two incidental findings were biparentally inherited likely pathogenic compound homozygous variant (nonsense variant in the *KPTN* gene) and maternal inherited likely pathogenic heterozygous variant (nonsense variant in the *TCF12* gene). The former was associated with autosomal recessive intellectual developmental disorder 41 (OMIM 615637), and the latter was associated with autosomal dominant craniosynostosis 3 (OMIM 615314). The two VUS were paternally inherited missense variants in the *COL1A1* gene related to autosomal dominant forms of osteogenesis imperfecta (OMIM 166210) and paternally inherited nonsense variant in the *COL10A1* gene related to autosomal dominant metaphyseal chondrodysplasia Schmid type (OMIM 156500). The absence of heterozygosity was also identified in eight chromosomes with approximately 173 Mb in size, which would raise the possibility of autosomal recessive disorders. Both parents were phenotypically normal. They were seen in the genetic clinic for counseling on family planning. In case GS2, skeletal abnormality in the fetus was associated with the pathogenic variant in the *COL1A1* gene. WGS also discovered two secondary findings in the father. One was a heterozygous pathogenic variant in the *LDLR* gene associated with autosomal dominant familial hypercholesterolemia (OMIM 143890). The other was a heterozygous pathogenic variant in the *G6PC* gene associated with autosomal recessive glycogen storage disease Ia (OMIM 232200). The family was counseled by the clinical geneticist to have family screening and regular medical check-ups of lipid profiles. Thus, the sequencing had an implication on reproductive genetic counseling in 10 cases.

**Table 2:** Results of whole exome sequencing.

Case	Gestational Age at Test Order	Prenatal Phenotype	Gene	Fetal Sequencing Result	ACMG Classification	Molecular Diagnosis	Inheritance	Novel Variant	Clinical Impact	Pregnancy Outcome	Post-Mortem Result/Postnatal Phenotype	Incidental Finding	Secondary Finding
ES1	Post-TOP	USG: absent bilateral lens, microphthalmia, left diaphragmatic hernia, overlapping fingers over bilateral hands	*RARB*	NM_000965.3:c.1210C>T p.(Gln404Ter), het, de novo	Likely pathogenic	Syndromic microphthalmia-12	Autosomal dominant	No	Information provision	TOP	Fused eyelids, absent lens of the left eye, left diaphragmatic hernia with herniation of the left lobe of the liver, the stomach, part of the small intestine and spleen into the left hemithorax	NR	NR
ES2	Post-TOP	USG: holoprosencephaly	*PPP1R12A*	NM_002480.2:c.609delp.(His204Thrfs*12), het, de novo	Pathogenic	Genitourinary and/or brain malformation syndrome	Autosomal dominant	No	Information provision	TOP	Gross examination showed no gross abnormality; declined autopsy	NR	NR
ES3	Post-TOP	USG: micrognathia, overriding aorta, ventricular septal defect, pulmonary stenosis	*NIPBL*	NM_015384.4:c.2642dup p.(Ser882Ilefs*10), het, de novo	Pathogenic	Cornelia de Lange syndrome 1	Autosomal dominant	No	Information provision	TOP	Mandibular hypoplasia, cleft palate, ventricular septal defect, overriding aorta, mild pulmonary stenosis, and Meckel’s diverticulum noted in the ileum	NR	NR
ES4	Post-TOP	USG: cystic hygroma, micrognathia, club feet, hydrops fetalis	*NEB*	NM_001164508.2:c.8712G>A p.(Trp2904Ter), het, mat NM_001164508.2:c.133_146delTCAGAAACTTCCAA p.(Ser45ThrfsTer48), het, pat	Likely pathogenic Likely pathogenic	Arthrogryposis multiplex congenita 6 Nemaline myopathy 2	Autosomal recessive	Yes No	Familial diagnosis, family planning and counseling	TOP	Gross examination found low-set ears, micrognathia, and club feet with abnormal posture; declined an autopsy	NR	NR
ES5	Post-TOP	USG: microcephaly, sloping forehead, flat occiput MRI: microcephaly, hypoplasia of bilateral frontal lobes and frontal horns, abnormal sulcation pattern with underdeveloped Sylvian fissure, suspected bilateral thalamic fusion and partial agenesis/dysgenesis of the corpus callosum	*ASPM*	NM_018136.4:c.349C>T, p.(Arg117*), homo, matpat	Pathogenic	Primary microcephaly 5	Autosomal recessive	No	Familial diagnosis, family planning and counseling	TOP	Gross examination found microcephaly, sloping forehead and flat occiput; declined fetal autopsy	NR	NR
ES6	16^+1^ weeks	USG: invisible cerebellum with dilated cisterna magna, cystic hygroma, upper and lower limbs in flexion posture, rocker bottom feet, hydrops fetalis	*HRAS*	NM_005343.2:c.182G>A p.(Gln61Arg), het, de novo	Pathogenic	Costello syndrome	Autosomal dominant	No	Information provision	Miscarriage at 18 weeks	Limited internal assessment because of extensive autolysis	NR	NR
ES7	17^+4^ weeks	USG: cystic hygroma, hydrops fetalis	*LZTR1*	NM_006767.3:c.848G>A, p.(Arg283Gln), het, mat	Variant of uncertain significance	Noonan syndrome	Autosomal dominant	No	Reproductive decision, familial diagnosis, family planning and counseling, and neonatal management and follow-up	Livebirth	Delivery at 38 weeks, at 2 months old, with clinical features of Noonan syndrome	NR	NR
ES8	Post-TOP	USG: occipital encephalocele, hypoplastic left heart, bilateral enlarged cystic kidneys, upper limbs and lower limbs short and bowed, feet with polydactyl	-	-	Negative	-	-	-	-	TOP	Encephalocele, hypoplastic thymus, hypoplastic of left heart, polycystic change of bilateral kidneys, feet with polydactyl	NR	NR
ES9	Post-TOP	USG: dysplastic mitral valve, atrioventricular septal defect, smallish distal aorta, persistent left superior vena cava, absent right kidney, ureterocele, single umbilical artery, persistent right umbilical vein	-	-	Negative	-	-	-	-	TOP	Gross examination found a 3 mm skin tag around the anus, no anal opening and pre-axial polydactyly (extra thumb) over left hand; declined fetal autopsy	NR	NR
ES10	Post-TOP	USG: small cerebellum, absent cavum septum pellucidum, small corpus callosum, mild hypertelorism	-	-	Negative	-	-	-	-	TOP	Mild hypertelorism, low-set ears and the marked autolytic change limited the assessment of internal structure of the brain	NR	NR
ES11	Post-TOP	USG: Heterotaxy syndrome with dextrocardia and central liver, right atrial isomerism, unbalanced atrioventricular septal defect, double outlet right ventricle, pulmonary stenosis, infracardiac total anomalous pulmonary venous connection, bilateral superior vena cava	-	-	Negative	-	-	-	-	TOP	Severe hypertelorism with prominent supra-orbital and infraorbital ridges, short nose, carp-sharped mouth, heterotaxy syndrome with dextrocardia and central liver, complex congenital heart disease with hypoplastic left ventricle, atrioventricular septal defect, bilateral superior vena cava, dominant right ventricle with small pulmonary artery, both lungs with 3 lobes and accentuation of other fissures	NR	NR
ES12	Post-IUD	USG: early onset intrauterine growth restriction, oligohydramnios, placentomegaly, single umbilical artery	-	-	Negative	-	-	-	-	Intrauterine death at 27^+4^ weeks	Weight 0.59 kg, low-set ears and receding jaw	NR	NR
ES13	Post-TOP	USG: bilateral hydrocephalus, small and abnormal cerebellum and deficient vermis, severe micrognathia, bilateral fixed equinovarus with pes cavus, left lower lobe congenital pulmonary airway malformations	-	-	Negative	-	-	-	-	TOP	Declined fetal autopsy	NR	NR
ES14	Post-TOP	USG: hydrops fetalis	-	-	Negative	-	-	-	-	TOP	Hydrops fetalis	NR	NR
ES15	Post-TOP	USG: alobar holoprosencephaly, depressed facial profile, no obvious nose seen, no proboscis, single orbit, hypoplastic left heart, left hand abnormal with second finger shorter and abnormal posture	-	-	Negative	-	-	-	-	TOP	Gross examination found a flat facial profile, absent nose, and shortened second finger of the left hand; declined fetal autopsy	NR	NR

Abbreviation: ACMG, American College of Medical Genetics and Genomics; Het, heterozygous; Homo, homozygous; Mat, maternal inherited; Matpat, biparental inherited; MRI, magnetic resonance imaging; NR, not reported; Pat, paternal inherited; TOP, termination of pregnancy; USG, ultrasound; * terminating.

**Table 3:** Results of whole genome sequencing.

Case	Gestational Age at Test Order	Prenatal Phenotype	Gene	Fetal Sequencing Result	ACMG Classification	Molecular Diagnosis	Inheritance	Novel Variant	Clinical Impact	Pregnancy Outcome	Post-Mortem Result/Postnatal Phenotype	Incidental Finding	Secondary Finding
GS1	32^+1^ weeks	USG: hydrocephalus, periventricular nodular heterotopia, mega cisterna magna, partial dysgenesis of the corpus callosum MRI: diffuse bilateral periventricular grey matter heterotropia, mega cisterna magna, normal corpus callosum	*FLNA*	NM_001456.3 exon39 c.6368_6369del p.S2123fs, hemi, mat	Likely pathogenic	Periventricular nodular heterotopia	X-linked	No	Familial diagnosis, family planning and counselling, neonatal management and follow-up	Livebirth	Delivery at 37^+1^ for IUGR, postdelivery MRI showed mild hydrocephalus, periventricular nodular heterotopia, prominent extra-axial cerebrospinal fluid spaces, mega cisterna magna, thinned corpus callosum; at 8 months old, normal growth and development	-	Fetus and father: heterozygous deletion on 16p13.3, including HBA1 and HBA2 (carrier of α thalassemia-1)
GS2	Post-TOP	USG: hypomineralized cranial bone above the base of the skull, short and angulated right femur	*COL1A1*	NM_000088.3 exon33 c.2299G>A p.G767S, het, de novo	Pathogenic	Osteogenesis imperfecta	Autosomal dominant	No	Information provision, familial diagnosis, family planning and counselling	TOP	Declined fetal autopsy	-	Father: heterozygous pathogenic variant, c.1747C>T(p.H583Y) in the LDLR gene (autosomal dominant familial hypercholesterolemia); carrier of pathogenic variant G6PC c.248G>A (autosomal recessive glycogen storage disease Ia)
GS3	Post-TOP	USG: agenesis of the corpus callosum, small cerebellum MRI: agenesis of the corpus callosum, small cerebellum	*SMARCB1* *LAMC3*	NM_003073.5 exon9 c.1130G>A p.R377H, het, de novo NM_006059.4 exon12 c.1963C>T p.R655W, het, mat	Pathogenic Variant of uncertain significance	Coffin-Siris syndrome Malformations of cortical development	Autosomal dominant Autosomal recessive	No No	Information provision, familial diagnosis, family planning and counseling	TOP	Gross examination found bilateral club foot with short big toes, bilateral clenched hands, low-set ears; declined fetal autopsy Placental pathologic finding of single umbilical artery	*LAMC3* NM_006059.4 exon18 c.3140G>A p.W1047X, het, pat (likely pathogenic, autosomal recessive malformations of cortical development)	-
GS4	Post-TOP	USG: semilobar holoprosencephaly, bilateral microphthalmia, left lens absent, hypotelorism, bilateral cleft lip and cleft palate, low thoracic scoliosis, suspected hemivertebra, bilateral club foot	*RERE* *GLI2*	NM_012102.4 exon19 c.3385C>G p.Q1129E, het, de novo NM_005270.5 intron13 c.2293+3G>A, het, pat	Likely pathogenic Variant of uncertain significance	Neurodevelopmental disorder with or without anomalies of the brain, eye, or heart Holoprosencephaly 9	Autosomal dominant Autosomal dominant	No	Information provision, familial diagnosis, family planning and counseling	TOP	Gross examination found bilateral microphthalmia, hypotelorism, bilateral cleft lips and cleft palate, micrognathia, ambiguous genitalia, and left clubfoot; declined fetal autopsy	-	-
GS5	Post-TOP	USG: Tetralogy of Fallot, persistent left superior vena cava, atrioventricular septal defect, horseshoe kidneys, single umbilical artery	*KMT2D*	NM_003482.4 exon11/55 c.1899dup p.Pro634AlafsTer7, het, de novo	Pathogenic	Kabuki syndrome	Autosomal dominant	Yes	Information provision	TOP	Tetralogy of Fallot with atrioventricular septal defect, horseshoe kidneys with fusion at the lower pole, single umbilical artery	-	-
GS6	29 weeks	USG: bilateral Sylvian fissure angle severely delayed, asymmetrical diagonal echogenic lines outside the ventricles, suggestive of neuronal migration disorder, aortic stenosis, aortopulmonary window, early onset symmetrical IUGR, oligohydramnios MRI: no structural brain abnormalities	*MAP2K2*	NM_030662.3 exon1 c.3G>A p.M1I, het, mat	Variant of uncertain significance	Cranio-facio-cutaneous syndrome	Autosomal dominant	No	Familial diagnosis, family planning and counselling, neonatal management and follow-up	Livebirth	Delivery at 35^+2^ weeks for abnormal Doppler and IUGR, patent ductus arteriosus with ligation for uncontrolled heart failure on day 20, bovine aortic arch, moderate aortic stenosis, secundum atrial septal defect, right renal stone; at 13 months old, gross motor delay on physiotherapy training, closed atrial septal defect, resolving nephrocalcinosis	-	-
GS7	Post-TOP	USG: hydrops fetalis	*COL1A1* *COL10A1*	NM_000088.3 exon47 c.3469G>A p.G1157S, het, pat COL10A1 NM_000493.4 exon3c.688C>T p.Q230X, het, pat	Variant of uncertain significance Variant of uncertain significance	Osteogenesis imperfecta Metaphyseal chondrodysplasia, Schmid type	Autosomal dominant Autosomal dominant	No No	Familial diagnosis, family planning and counselling	TOP	Gross examination found hydropic abortus; declined fetal autopsy	*KPTN* NM_007059.4 intron4 c.450-2A > G, hom, matpat (likely pathogenic, intellectual developmental disorder, autosomal recessive 41) *TCF12* NM_207036.2 exon11 c.956C>G p.S319X, het, mat (likely pathogenic, autosomal dominant, craniosynostosis 3) 172.8 Mb of the absence of heterozygosity region	-
GS8	21^+4^ weeks	USG: micrognathia	-	-	Negative	-	-	-	Reproductive decision	Livebirth	Delivery at 39^+5^ weeks, normal newborn examination	-	Fetus and father: heterozygous deletion on 16p13.3, including HBA1 and HBA2 (carrier of α-thalassemia-1)
GS9	20^+3^ weeks	USG: megacystis, bilateral dilated renal pelvises	-	-	Negative		-	-	Reproductive decision	Livebirth #	Delivery at 39^+3^ weeks, at 5 months old, bilateral grade 4 vesicoureteral reflux, posterior urethral valve with bilateral hydronephrosis	-	-
GS10	19^+2^ weeks	USG: megacystis, bilateral dilated renal pelvises, umbilical cord cyst oligohydramnios	-	-	Negative		-	-	Reproductive decision	TOP	Termination of pregnancy in the private sector, postmortem report not available	-	-
GS11	Post- selective TOP 20^+2^ weeks	USG: monochorionic, diamniotic twin, with one hydrops with cystic hygroma	-	-	Negative		-	-	Reproductive decision	Selective feticide; co-twin livebirth	Delivery of remaining twin at 41 weeks, normal newborn examination	-	-
GS12	Post-TOP	USG: cerebellar hypoplasia, cardiomegaly, right ventricular hypertrophy, pericardial effusion	-	-	Negative		-	-	-	TOP	Cerebellar hypoplasia, increased right cardiac ventricular wall thickness, atrial septal defect, pulmonary hypoplasia		
GS13	Post-TOP	USG: cerebellar hypoplasia, left ventriculomegaly	-	-	Negative		-	-	-	TOP	Hypertelorism, left ventriculomegaly, inconclusive of cerebellar hypoplasia, right thumb hypoplasia		
GS14	Post-TOP	USG: bilateral borderline ventriculomegaly, agenesis of the corpus callosum MRI: complete agenesis of the corpus callosum	-	-	Negative		-	-	-	TOP	Agenesis of corpus callosum		

Abbreviation: ACMG, American College of Medical Genetics and Genomics; Hemi, hemizygous; Het, heterozygous; IUGR, intrauterine growth restriction; Mat, maternal inherited; MRI, magnetic resonance imaging; Pat, paternal inherited; TOP, termination of pregnancy; USG, ultrasound. # Chromosomal microarray analysis revealed a paternally inherited variant of uncertain significance (copy number gain of chromosome 18q11.1-q11.2). The final karyotyping result was supernumerary ring chromosome 18 syndrome inherited from the father, who also had a history of ureteroplasty and reimplantation of the ureter into the bladder as a teenager.

### 3.4. Personnel Involving in Pre-Test and Post-Test Genetic Counselling

The majority of cases received pre-test genetic counseling conducted by a maternal-fetal medicine specialist or obstetrician with genetic expertise (27/29, 93.1%). In post-test genetic counseling, clinical geneticists were involved in 10 cases (10/29, 34.5%), including all cases with uncertain results, cases with incidental/secondary findings, cases with inherited causative variants except for one case in which the parents returned to their own country and could not attend the post-test counseling.

## 4. Discussion

Our first-year experience of application of advanced sequencing technologies in public healthcare service for prenatal diagnosis of fetal structural anomalies achieved a diagnostic yield of 37.9%. Our diagnostic yield is similar to the recent review of worldwide studies [7]. The latest systematic review and meta-analysis of 66 cohort studies with WES/WGS on prenatal samples for fetal anomalies, including 4350 anomalous fetuses from 2010 to 2021, showed the pooled incremental yield of genomic sequencing after normal karyotype/CMA was 31% (95% confidence interval 26–36%) [7]. In addition, subgroup analysis found a significantly higher incremental yield with selected cases for more likely monogenic disease compared to unselected cases (42% vs. 15%). Locally, a multidisciplinary team comprising 15 health care professionals in different specialties was organized to help with case selection in this public funded service. The setup of a web-based platform allows convenient access to anonymous medical data of submitted cases, including ultrasound images, promotes sharing of information and creates more interaction and communication among the multidisciplinary team members. We select the cases with reference to the updated eligibility criteria from overseas programs (e.g., National Health Service (England) [25]) and professional society recommendations [10,11]. With the emergence of more and more literature about prenatal presentations of genetic disorders, the selection criteria are also widened to include a fetus with increased nuchal translucency (≥6.5 mm) plus another anomaly and isolated non-immune fetal hydrops [25,26,27,28,29]. Besides, the diagnostic yield may also vary widely across different structural abnormalities, as previous studies reported higher yields in multisystem anomalies, isolated skeletal anomalies and isolated brain anomalies [7]. Despite our relatively small sample size, these phenomena were also observed in our highly selected cohort.

We retrospectively assessed the impact of prenatal genomic sequencing on clinical management in our service review. Overall, the clinical impact of receiving a genomic sequencing result was observed in 55.2% (16/29) of the cases, which influenced reproductive decision-making in four cases, guided perinatal management in two cases and helped future family planning in ten cases. Although the majority of our cases were post-abortion pregnancies, knowing the definitive genetic diagnosis for a fetus with ultrasound abnormalities assisted in better recurrence risk assessment in 10 families. Detection of de novo variants may alleviate the guilty feeling for the parents and provide reassurance of low recurrence risk in their subsequent pregnancies, although it is not negligible because there may be a possibility of germline mosaicism and other genetic cause that cannot be totally excluded. On the other hand, the detection of inherited variants can alter reproductive planning and allow prenatal testing or in-vitro fertilization with preimplantation genetic diagnosis in future pregnancies. Molecular genetic test also allows more accurate prognostic prediction, which is very important for the parental decision-making process about pregnancy continuation. A significant impact was observed in our four cases, and the parents continued the pregnancy after knowing no causal variants were identified, and most monogenetic disorders were excluded. In addition, definite genetic results can guide obstetric and neonatal management. For example, the non-diagnostic sequencing report in case GS6 helped adjustment the antenatal surveillance and the time of delivery of the severe early-onset growth-restricted fetus at later gestation until abnormal ultrasound Doppler occurred. Another example, case GS1, was a female fetus with a structural brain malformation due to an inherited pathogenic variant of the *FLNA* gene. It is known as periventricular nodular heterotopia, which is a neuronal migration disorder with an X-linked dominant mode of inheritance [30]. Heterozygous females can be asymptomatic and have normal intelligence, but some may suffer from seizures and various cardiovascular manifestations. Hemizygous males tend to die in utero. The mother and her first daughter were assessed by a clinical geneticist, and her newborn was then under pediatric follow-up. Therefore, a prenatal genetic diagnosis can trigger the cascade screening in relatives of probands, shorten the postnatal diagnostic odyssey and facilitate personalized medical treatment and follow-up for the baby after birth without delay. Furthermore, other studies also demonstrated the identification of a genetic disorder in the prenatal period could have additional benefits that helped reduction of maternal morbidity by avoiding operative delivery for a fetus with a poor prognosis and improved the baby’s quality of life by withholding futile and painful life-sustaining procedures and arranging palliative care [21,22,24]. The broadening of prenatal genetic diagnostic capabilities can also help in the selection process for fetal surgery and open the potential target for in-utero treatment [31]. The ultimate goal of genetic diagnosis is to prevent and reduce morbidity and mortality.

The development of high-throughput next-generation sequencing allows the large-scale and rapid assessment of entire genomes. However, it introduces challenges with respect to the interpretation of genetic variation and communication of the uncertain genetic findings to the family [32]. In our cohort with the application of WES and WGS technologies, six VUS were found in 5 fetuses, yielding an overall VUS detection rate of 17.2% (5/29), 6.7% (1/15) for WES and 28.6% (4/14) for WGS. A systematic review by Pratt et al. reported VUS rate of exome sequencing for fetuses with structural anomalies ranged from 3.9% to 20% [33]. Exome sequencing examines only the protein-coding regions (exons), which constitute 1%–2% of the genome but harbor more than 85% of all disease-causing mutations, whereas whole genome sequencing examines the whole genome. Potentially WGS can increase the sensitivity of diagnostic variant detection because of comprehensive DNA coverage, including the non-protein-coding regions (introns) and structural variant/copy number variants; however, it could lead to increased costs, increased frequency of VUS and decreased interpretability. In our study, the VUS rate of our WGS cohort was higher compared with our WES cohort. Genomic sequencing is a phenotype-driven test [11]. The interpretation and classification of genetic variants is a complex procedure that requires bioinformatics support to weigh evidence from prediction tools, population frequency, co-occurrence, segregation, and functional studies [34]. In the prenatal setting, the interpretation is more complicated as we lack publicly available prenatal ultrasound-genotype databases. The causal relationship can be difficult to delineate because of possible coincidental findings. In addition, the accuracy and completeness of prenatal phenotyping are limited by prenatal ultrasound resolution and scanning techniques; the prenatal features are sometimes non-specific and may change with gestation. Further fetal imaging strategies, such as fetal MRI, can play an important adjunctive role in confirming or excluding abnormal findings detected by ultrasound, especially for brain abnormalities. The benefits of its use were demonstrated in our cases GS1, GS3, GS6 and GS14. For terminated fetuses or deceased fetuses/neonates with prenatal identified structural abnormality, detailed postmortem examination can supplement additional information on subtle dysmorphic abnormalities not visualized in prenatal imaging, for example, in our cases ES11 and ES12. All can maximize and refine the phenotypic information to improve genetic variant interpretation. Our case ES7 illustrated the difficulties that can occur in interpreting genotype-phenotype causality during the prenatal period. The hydropic fetus harbored a maternally inherited variant in the *LZTR1* gene, which mutated gene is associated with autosomal dominant Noonan syndrome. After discussion among the laboratory scientists, geneticists and fetal-medicine specialists, the variant was reported as VUS because of conflicting data from public variant databases and a lack of prenatal phenotypic information about this variant. The mother was apparently normal, and the family received detailed post-test counseling with a clinical geneticist. Finally, the pregnancy was continued, and her baby was born with features of Noonan syndrome and a subsequent pediatric assessment was arranged. Therefore, the variant inherited from a normal parent may provide false reassurance to the parents and healthcare providers. But genetic diseases can have a wide spectrum of clinical presentations and variable outcomes due to incomplete penetrance and variable expressivity. Hence, it is important the additional postnatal phenotypic information of the proband can be returned to the laboratories as well as to public databases for the re-analysis of its pathogenicity. Re-classification of genetic variants to benign or pathogenic can occur over time as more experience and scientific evidence on new genotype-phenotype correlation evolves. Two studies reported the reclassification of variants in their cohorts after birth and even a few years later [22,35]. This future revision of data and data sharing will be beneficial to the parents as they may request prenatal or preimplantation genetic diagnosis to avoid recurrence, as well as counseling for future parents encountering the same genetic variant. After all, the uncertain finding is unavoidable, and it can create significant parental anxiety as well as difficulties in information processing and decision-making. Therefore, it is essential to prepare them psychologically by telling them about the possibility of finding and reporting VUS before taking the test [36].

Compared with single-gene and panel tests, genome-wide DNA sequencing increases the chance of reaching a definite genetic diagnosis; however, it also increases the possibility of identifying incidental and secondary genetic findings in a fetus and/or parents. In our review, we found three incidental findings in two fetuses, a heterozygous likely pathogenic variant in *LAMC3* with a heterozygous VUS in the same gene in case GS3; two homozygous biallelic likely pathogenic variants in the *KPTN* gene and a heterozygous likely pathogenic variant in the *TCF12* gene together with the absence of heterozygosity in case GS7. These pathogenic variants were all inherited from the parents and are associated with neurodevelopmental disorders having incomplete penetrance. Some deleterious recessive genes may also be hidden in the loss of heterozygosity chromosomal region. In view of the possible implications for reproductive planning, these findings were reported to the parents as they opted to receive incidental findings in the pre-test consent process. The father of case GS2 also received secondary findings about his carrier status of two genetic conditions. Currently, there is no universal consensus on reporting incidental and secondary findings which are unrelated to the indication for sequencing in a prenatal setting. The disclosure is debatable as it carries potential clinical and ethical challenges apart from medical benefits to the family [37]. Some of the clinical challenges described included workload in counseling, diagnosis confirmation, screening, and interventions, together with raised parental anxiety and social stigmatization. The ethical issues included pregnancy termination based on the secondary findings and the unborn child’s autonomy. Some guidelines issued by professional bodies recommend parents should know the reporting policy and have the option to opt-out of revealing this information in the informed consent process after consideration of the possible implication to them and others in the family after positive results [10,11]. Parents should also be aware of the additional findings of non-paternity or consanguinity.

Furthermore, all parents undergoing sequencing are recommended to receive post-test counseling conducted by individuals with relevant genetic expertise, irrespective of the result findings [10,11]. All our cases with uncertain, positive and incidental/secondary genetic findings received post-test counseling provided by clinical geneticists who could help in the provision of accurate information based on current knowledge and non-directive supportive counseling tailored to the genetic literacy of each individual. It is of utmost importance as the reproductive decision is based on the understanding of the information given as well as personal values. Apart from counseling on genetic etiology, fetal structural anomalies have to be managed after birth by different parties, such as neontologists, pediatricians, cardiologists, and surgeons. For example, our cases GS9 and GS10 having fetal urinary tract abnormality would be highly beneficial by talking to a urologist about the child’s long-term renal prognosis, the options of renal support and urological interventions for potential urinary obstruction and vesicoureteral reflux. Therefore, multidisciplinary counseling by knowledgeable health professionals on treatment options and prognosis covering all aspects of their baby’s physical anomaly should be offered, preferably supplemented by written, visual and web-based resources [38]. This would help reduce parental anxiety. Moreover, psychological stress after fetal diagnoses should be addressed, and emotional support should be provided throughout the pregnancy or termination procedure [39,40].

Among our eight ongoing pregnancies undergoing the genomic sequencing, the median turnaround time of the genetic report was 19.5 days (average, 20.4 days; range, 13–31 days), which was similar to the meta-analysis of 15 studies by Mellis et al. reported the median turnaround time for exome sequencing of 20 days (range, 4–141 days) [7]. A faster turnaround time for prenatal diagnosis is important because of the reproductive decision within the local legal time limit of pregnancy termination and the influence on immediate neonatal management. This would depend on the technical workflow, DNA sequencing approaches, data interpretation pipeline, reporting policy and complexity of the genetic findings. Trio analysis can help fasten the bioinformatics analysis [31]. Importantly, the pre-test counseling should address the expected timeframe of the result’s return to the local testing laboratory.

A major limitation of this study is the small sample size. This is a balance between careful case selection based on professional society guidelines, resource allocation and the local laboratory capability. Despite this limitation, our findings constitute a good estimate of genome-wide sequencing yield on prenatal diagnosis for fetal anomalies. Since this clinical service is ongoing, a larger sample size with follow-up information would be available in a future review. The VUS rate and unsolved cases would be helped by future data re-analysis. Another limitation is the different sequencing techniques and reporting policies used by two local prenatal laboratories. Prenatal exome sequencing is predominantly used in clinical practice and research. Prenatal genome sequencing is an emerging technology, and there are limited studies on its prenatal application. With its potential to detect kinds of genetic variation, genome sequencing may replace chromosomal microarray and exome sequencing in the future. Although our review is not a direct comparative study between WES and WGS, it would give us some insight into the clinical benefits and limitations of different sequencing approaches. On the other hand, the widespread use of genome-wide sequencing technology would carry resource implications in overall health economics as well as socio- ethical concerns. The challenges would be further heightened if it is used in non-invasive approaches or moved into the commercial market. It is imperative for the usage of prenatal genomic testing to be well-regulated to ensure good clinical ethics. Future prospective studies are required to evaluate the psychological impact of the molecular diagnosis on the parents and the cost-effective analysis of next-generation sequencing applications in prenatal diagnostics.

To our knowledge, this is the first evaluation study to look at the public prenatal sequencing service in real clinical practice in Hong Kong. We demonstrated the feasibility as well as the clinical utility of the new prenatal WES/WGS service to identify the genetic diagnosis for fetal structural anomalies after normal conventional tests. In addition, two novel genetic variants were discovered. The new genetic findings can help to understand human fetal development and reveal the unrecognized prenatal phenotypes associated with monogenetic diseases. Our service is under the public-funded framework with a stringent selection process and clear inclusion criteria so that everyone can access the free clinical service fairly without deprivation associated with poverty.

## 5. Conclusions

Our review supports the important role of genome-wide sequencing services in the prenatal diagnosis of fetal structural anomalies in a population setting. Involving a multidisciplinary team approach in case selection, variant interpretation, counseling on genetic aspects and perinatal management, and the provision of future care to the unborn child are essential to support a comprehensive genetic service.

## Figures and Tables

**Table 1:** Basic patient characteristics, fetal phenotypic features, genetic results and pregnancy outcomes.

Characteristic	Value *
Ethnicity	
Chinese	26 (89.7)
South Asian	3 (10.3)
Maternal age (year)	33 (25–39)
Educational level	
Primary	0 (0)
Secondary	9 (31.0)
Tertiary	18 (62.1)
Unknown	2 (6.9)
Married	28 (96.6)
Consanguinity	2 (6.9)
Family history of genetic problems	1 (3.4)
Previous pregnancy/child of genetic problems	2 (6.9)
Nulliparity	19 (65.5)
History of pregnancy loss	6 (20.7)
History of pregnancy termination	4 (13.8)
Planned pregnancy	23 (79.3)
Conception	
Natural	26 (89.7)
Assisted	3 (10.3)
Number of fetuses	
Singleton	28 (96.6)
Twin	1 (3.4)
Type of testing	
Whole exome sequencing (WES)	15 (51.7)
Whole genome sequencing (WGS)	14 (48.3)
Trio analysis	29 (100)
Invasive diagnostic procedure	
No	3 (10.3)
Chorionic villus sampling	7 (24.1)
Amniocentesis	19 (65.5)
Source of fetal DNA	
Chorionic villi	6 (20.7)
Amniocytes	19 (65.5)
Products of conception	3 (10.3)
Fetal blood/tissue	1 (3.4)
Prenatal phenotype at test request	
Single system	11 (37.9)
- Brain malformation	7 (24.1)
- Renal malformation	2 (6.9)
- Skeletal malformation	1 (3.4)
- Facial malformation	1 (3.4)
Multiple systems	11 (37.9)
Intrauterine growth restriction	1 (3.4)
Hydrops fetalis	6 (20.7)
Timing of test request	
Before 24 weeks of pregnancy	6 (20.7)
After 24 weeks of pregnancy	2 (6.9)
After intrauterine death	1 (3.4)
After the termination of pregnancy	20 (69.0)
Report turnaround time (day) during pregnancy	19.5 (13–31)
WES results (genetic variant)	
Pathogenic/Likely pathogenic	7 (46.7)
Variant of uncertain significance	1 (6.7)
Negative	8 (53.3)
WGS results (genetic variant)	
Pathogenic/Likely pathogenic	5 (35.7)
Variant of uncertain significance	5 (35.7)
Negative	7 (50.0)
Incidental finding	3 (21.4)
Secondary finding	4 (28.6)
Pregnancy outcome	
Livebirth	5 (17.2)
Intrauterine death	1 (3.4)
Miscarriage	1 (3.4)
Termination of pregnancy	22 (75.9)
Neonatal death	0 (0)
Personnel conducting pre-test counseling	
Midwife	0 (0)
Maternal-fetal medicine specialist/obstetrician	27 (93.1)
Clinical geneticist	2 (6.9)
Personnel conducting post-test counseling	
Midwife	1 (3.4)
Maternal-fetal medicine specialist/obstetrician	17 (58.6)
Clinical geneticist	10 (34.5)
Not available	1 (3.4)

* Data are presented as median (range) or No. (%) of patients.

## Data Availability

The datasets used and analyzed during the current study are available from the corresponding author upon reasonable request.
